# On the Use of Glycerine as an Internal Remedy

**Published:** 1855-03

**Authors:** J. L. Crawcour

**Affiliations:** New Orleans


On the use of Glycerine as an Internal Remedy.—By J. L. Crawcour, M. D., of New Orleans.—I wish to draw attention, particularly at present, to the *special* action of glycerine on the economy, and the perfect safety with which it can be used as an internal remedy. For the past twelve months I have used it in every case of disease where formerly I should have used cod liver oil, and with superior benefit; for while it seems to possess all the remedial virtues of this latter agent, it is its superior in taste, in not disordering the digestion, and in its property of combining with any other remedy.

In several cases of phthisis, of scrofulous disease generally, in mesenteric disease in children, I have used it largely and successfully; and in children, its sweet and agreeable taste gives it a great advantage over cod liver oil, the only agent I can compare it with, in its therapeutic action. In addition to its anti-strumous property, I find that it materially aids in the assimilation of salts of iron, especially of the iodide, and I now rarely order either iodine, or the iodide of iron, without combining them with glycerine. Quinine also, is soluble in it, without the aid of sulphuric acid, and to some slight extent is divested of its bitterness.

The dose in which I usually administer it is from one to three drachms three times daily, in an ounce of water; in from one to two drachms, it, in a short period, relieves the cough, improves the digestive powers, and appears to increase the fat producing principle in phthisical patients; in larger doses it has in a few instances produced nausea; it is, however, essentially necessary to its successful employment that it be obtained pure, and this is a matter of some difficulty, for it is ordinarily the result of the preparation of the common lead plaster, and consequently contains traces of lead, but by the process of Dr. Morfit, who decomposes lard, or oil, with hydrate of lime, it can be procured chemically pure, and at a very cheap rate. Should my communication induce other physicians to try it, the purpose of my writing to you will be answered, as it will be an equal boon to the physician and patient, if a remedy can be discovered equal in properties to cod liver oil and without its nauseous taste and smell.

I have recently tried it as a solvent for phosphorus, which latter we have hitherto hardly been able to use in medicine, as its solution in oil is so nauseous that we can rarely induce patients to swallow it, and its solution in ether is so dangerous that I question whether any physician of ordinary prudence would prescribe it. But in glycerine, it is not only nearly as soluble as in oil, but is miscible with water in all proportions, and is comparatively tasteless and odorless. About two grains dissolve readily in an ounce of boiling glycerine, and from experiments on myself, I consider it a powerful and a valuable stimulant. Of the above solution, which, after the nomenclature of Messrs. Cap and Garot, I would call the glycerole of phosphorus, I took one drachm in a wine-glassful of water, with which it intimately blended; there was hardly any taste or smell, and it did not produce those garlicky eructations said to be the result of the phosphorus in oil. Its effect on my system was that of a stimulant: in about half an hour the pulse became quickened, both at the wrist and at the temples, the cheeks flushed, the skin became warm and suffused with moisture, there was a certain amount of mental excitement, and after a short time there occurred a feeling of oppression at the præcordia, accompanied by palpitation of the heart, while after a certain time the cerebral excitement was followed by a slight feeling of confusion, accompanied by sleeplessness. Each time that it has been taken in this dose, it has, on myself, produced these same effects. I therefore look upon this quantity as excessive, and would suggest that if this remedy be used, it should be in doses ranging from ten to thirty minims. It may be combined with any other drug, and I look upon it as a valuable addition to our list of therapeutic agents; not that the use of phosphorus is new, but hitherto it has been excluded from us from the difficulty of finding a proper solvent—this now, I believe, has been discovered in glycerine.—*New Orleans Med. News & Hospital Gazette.*

